# Sources of Secondary Metabolite Variation in *Dysidea avara* (Porifera: Demospongiae): The Importance of Having Good Neighbors

**DOI:** 10.3390/md11020489

**Published:** 2013-02-18

**Authors:** Sonia De Caralt, Delphine Bry, Nataly Bontemps, Xavier Turon, Maria-Jesus Uriz, Bernard Banaigs

**Affiliations:** 1 Center for Advanced Studies of Blanes (CEAB-CSIC), Accés a la Cala St Francesc 14, 17300 Blanes, Girona, Spain; E-Mails: xturon@ceab.csic.es (X.T.); iosune@ceab.csic.es (M.-J.U.); 2 Environmental and Biomolecular Chemistry Laboratory, University of Perpignan Via Domita, 52 Paul Alduy Ave., Perpignan Cedex 66860, France; E-Mails: delphine.bry@hotmail.fr (D.B.); bontemps@univ-perp.fr (N.B.); banaigs@univ-perp.fr (B.B.)

**Keywords:** secondary metabolites, chemical ecology, sponges, temporal variation, intra-individual variation

## Abstract

Several studies report temporal, geographical, and intra-individual variation in sponge metabolite yields. However, the internal and/or external factors that regulate the metabolite production remain poorly understood. *Dysidea avara* is a demosponge that produces sesquiterpenoids (avarol and derivatives) with interesting medical properties, which has prompted addressed studies to obtain enough amounts of these metabolites for research on drug discovery. Within this framework, specimens of *Dysidea avara* from apopulation of the Northwest Mediterranean were sampled and their secondary metabolites quantified to assess their variability and the possible relationship with external (seasonality, interactions with neighbors) and internal (reproductive stages) factors. The results show a variation of the amount of both avarol and its monoacetate derivative with time, with no clear relationship with seawater temperature. A trade-off with sponge reproduction was not found either. However, our results showed for the first time that sponges are able to increase production or accumulation of secondary metabolites in their peripheral zone depending on the nature of their neighbors. This finding could explain part of the high variability in the amount of secondary metabolites usually found in chemical ecology studies on sponges and opens new biotechnological approaches to enhance the metabolite yield in sponge cultures.

## 1. Introduction

A great many of today’s commonly used medicines have arisen from naturally produced metabolites. Pharmaceutical research has developed important plant-derived drugs and in recent years the sea has become a new source of natural products. Marine invertebrates, especially sponges, are a prolific source of novel secondary metabolites with pharmacological applications [1,2].

In the framework of chemical ecology, many studies targeting marine sponges have established parallels with studies on plants in terrestrial systems. Both plants and sponges are important contributors, in terms of biomass and interactions, to their communities [3,4]. Both are sessile organisms whose ecological interactions (such as defence against predators, foulers, and competitors for space) are often chemically-mediated through secondary metabolites (reviewed in [5,6,7]). Grazing may produce similar harmful effects on plants and sponges; the plant response against the epiphytes can be compared to the antifouling reaction of some sponges; while the plant-plant interactions are equivalent to the interactions occurring during space competition among invertebrates and algae in marine benthic systems.

Despite these similarities, the high variability in the production of secondary metabolites at several levels seems to be a particularity of marine sponges [8,9,10,11]. Different types of factors have been reported to modulate the production of secondary metabolites in marine sessile organisms, both abiotic (salinity, pollutants, light, and temperature) and biotic (space competition, predation, fouling, and presence/absence of bacterial symbionts) [6,12,13,14]. However, studies of the patterns and causes of this variation in relation to biological and/or ecological factors lag far behind those on terrestrial plants [15,16].

Studies on the temporal variation in the production of bioactive metabolites in sponges have reported contradictory results. In some cases, the temporal variation correlated with temperature [8,17,18,19] and/or the reproductive cycles [20,21,22]. However, no clear temporal trend has been evidenced in other cases [18]. Even more puzzling, contrasting temporal patterns have been found for the same metabolites in sponges from different geographical locations [23]. Thus, no general conclusions can be drawn at present about the causes of temporal variation of secondary metabolites in sponges.

Spatial variation of sponge secondary metabolites has been reported to occur on several scales: Between localities [8,10,11] and within localities in contrasting habitats differing in the amount of irradiance [15] or predation pressure [4]. Furthermore, variation has also been reported at the intra-individual level in sponges, with several studies addressing either the identification of the cell types responsible for the metabolite biosynthesis or storage [24,25,26,27,28] or the metabolite concentration in several sponge zones [19,20,29,30,31,32,33], generally finding higher concentrations of the target metabolites at the sponge periphery than in the center. The preferential accumulation of the bioactive metabolites in the peripheral or apical parts of the sponges has been attributed to a defensive role against foulers [30] or predators [19,29,30,31,32,34]. Much less attention has been paid to the role of secondary metabolites in space competition [20,35,36,37], and this issue deserves further attention.

In spite of the above mentioned studies, the sources of the high variation in the production of secondary metabolites in sponges are not fully understood. New approaches are necessary to identify the factors (biotic and abiotic) that modulate metabolite production. Knowledge of the driving factors is of broad interest from both ecological and biotechnological points of view, as this knowledge can be used to enhance secondary metabolite production in target species, thus alleviating the supply problem faced when attempting technological applications [9,38].

*Dysidea avara*, Schmidt 1862, is a common Mediterranean sublittoral demosponge that produces the sesquiterpene hydroquinone avarol and its quinone derivative avarone in high amounts [39], along with minor derivatives [40]. Avarol has diverse medical activities such as anti-leukemic, anti-cancer, or anti-psoriasis properties, among others [41,42,43,44]. The huge biomedical potential of avarol has prompted a number of investigations focused on obtaining a supply for the pharmaceutical industry, based either on its synthetic production [45] or on *D. avara* cultivation [46,47,48].

The aim of this work was to study temporal and intra-individual variability of avarol yields in a population of *Dysidea avara* from the NW Mediterranean Sea and search for biotic and abiotic factors related to this variability. The rationale was to identify periods of the year and ecological situations where production of avarol was maximized, to optimize culture strategies for obtaining this compound. A two-year-long survey on avarol concentration was done to assess temporal patterns possibly related to temperature variation or to the sponge reproductive cycle. We also considered other external factors, such as the sponge interactions with neighbors, which may modulate the avarol concentration. We anticipated that metabolite concentration in the central part of the sponge was driven primarily by internal (intrinsic) factors, while the amount of metabolite in the sponge periphery could be influenced by external, biotic factors (e.g., space competition). Thus, we studied avarol yields in the central *vs.* peripheral parts of the sponges, as a function of the type of neighboring organism (either algae or invertebrates).

## 2. Results

### 2.1. Temporal Variation of Metabolites

**Figure 1:**
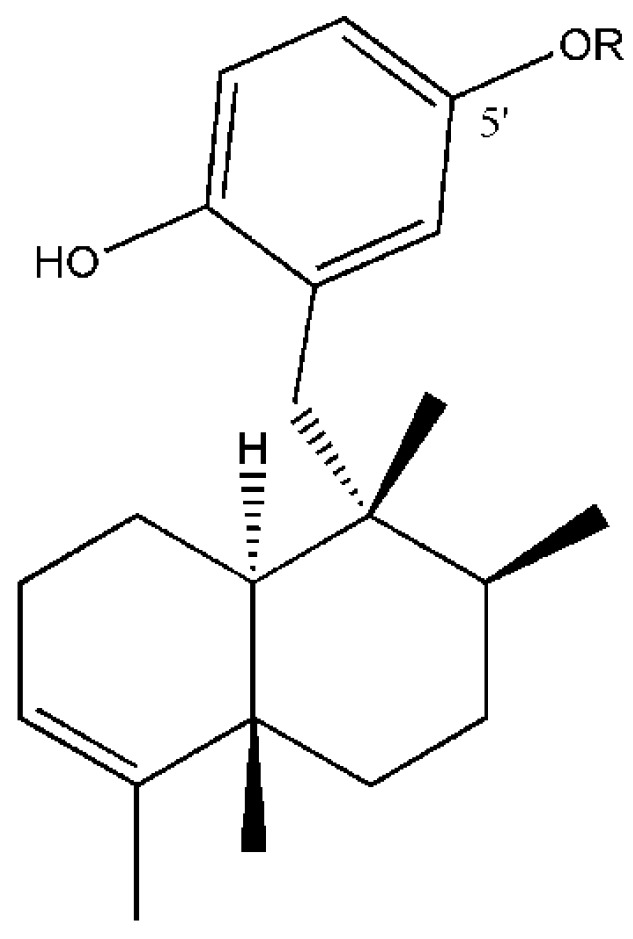
Structures of compounds from *D. avara*: Avarol (R = H) and 5′-monoacetylavarol (R = COCH_3_).

The qualitative analysis showed that all samples contained avarol (sesquiterpene hydroquinone) as the major compound and the monoacetylated derivate 5′-monoacetylavarol [40] as a minor compound (Figure 1). Although avarone (sesquiterpene quinone derivative of avarol) has been described as one of the main secondary metabolites in *D. avara* [40,49], we only found traces of avarone in some samples.

**Figure 2:**
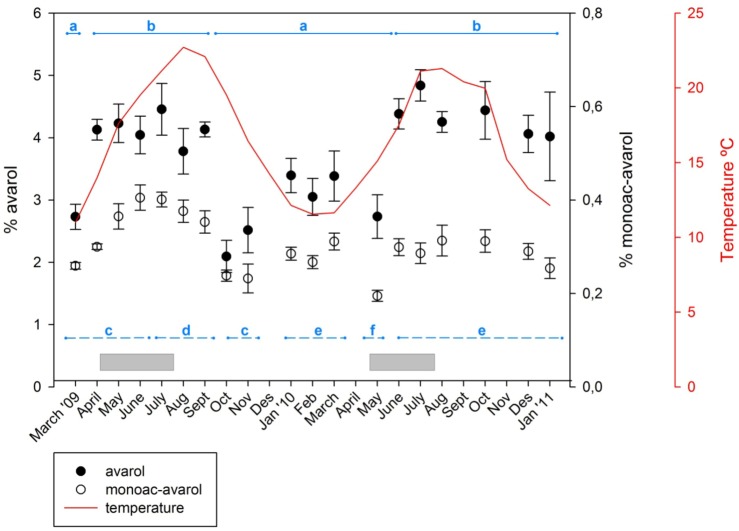
Temporal variation of avarol and 5′-monoacetylavarol. Percentage of avarol and 5′-monoacetylavarol (mg of the secondary metabolite/mg of sponge dry weight) over 23 months. Vertical lines are standard errors. Continuous line depicts the temperatures recorded at the study site. Horizontal bars (in grey) represent the extent of the reproductive period of *D. avara* at the study site. The horizontal lines show the statistically significant differences among months with PERMANOVA (solid line for the avarol and dashed line for the 5′-monoacetylavarol).

The time course of the concentrations of the two metabolites over the study period is shown in Figure 2. Over the temporal survey, the concentrations of avarol found in *Dysidea avara* ranged from 2.09% to 4.83% (relative to sponge dry weight) with an average of 3.68% ± 0.174% (SE); the concentration of 5′-monoacetylavarol ranged from 0.195% to 0.405% with an average of 0.302% ± 0.013% (SE). Over the study period the amount of 5′-monoacetylavarol represented a percentage of the avarol in the sample, ranging from 9.9% to 16.2% (13.7% ± 0.477%, mean ± SE).

Both compounds followed approximately the same seasonal pattern with higher concentrations in spring–early summer and lower values in fall–winter. Although the highest amounts of avarol and 5′-monoacetylavarol were reached in June–July of both years, the pattern was less evident in the second year. The most significant difference between the two monitored years was a minimum of avarol production found in October–November 2009, which did not occur in 2010.

Significant differences between months were found for both metabolites (Permutational multivariate analysis of variance (PERMANOVA), *p* < 0.001) although multiple comparisons showed that differences in one metabolite were not mirrored by the other compound (Figure 2). For avarol, periods of low and high avarol concentration were alternating. Higher avarol values were recorded from April to September 2009 and from June 2010 to January 2011. The lowest values of avarol were recorded in October and November 2009, followed by low values until May 2010. The 5′-monoacetylavarol had high yields from June to September 2009 and low values in October and November 2009, and in May 2010. The among-months variation for both metabolites (coefficient of variation was 0.206 and 0.190 for avarol and 5′-monoacetylavarol, respectively) was lower in most cases than the monthly between-individual variation (mean coefficient of variation was 0.25 and 0.214 for avarol and 5′-monoacetylavarol, respectively).

**Figure 3:**
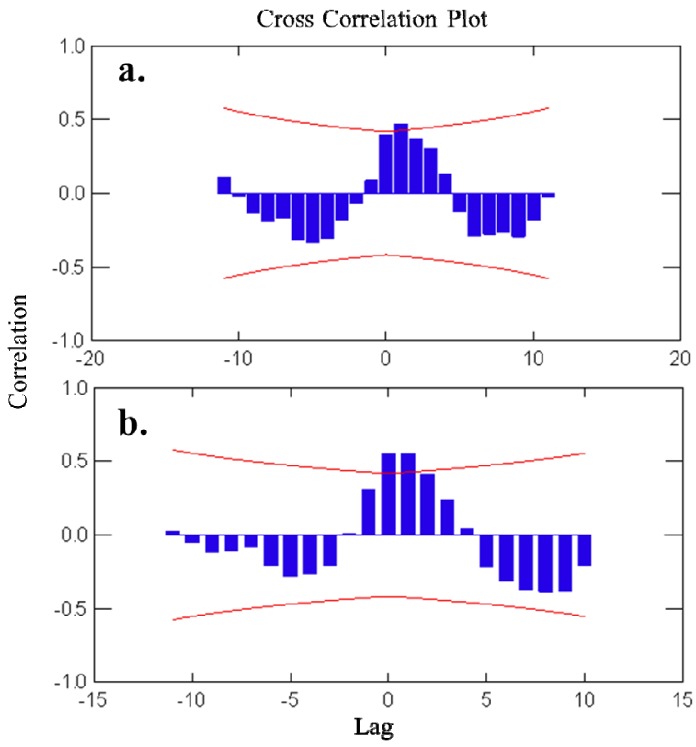
(**a**) Cross-correlation analyses of avarol concentration *versus* temperature. (**b**) Cross-correlation analyses of 5′-monoacetylavarol concentration *versus* temperature. Bars represent correlation coefficients between time series lagged at a specific number of months (Lag). Curved lines represent 95% confidence intervals of the correlation coefficients.

Autocorrelation plots (Figure 3) showed a pattern of seasonally fluctuating values, with positive and negative correlation coefficients alternating at lag-periods of *ca.* 6 months. For avarol, a significant correlation was found with the temperature readings in the subsequent 3 months (+1 to +3 months time-lag). For monoacetyl avarol, a positive and significant correlation was found at time-lag 0 and time-lags +1 and +2 (*i.e.*, with temperature of the two subsequent months). The fact that changes in temperature follow (positive lags) rather than precede changes in metabolite concentration indicated that temperature *per se* is not responsible for the changes in avarol and 5′-monoacetyl avarol production over the year. The brooding period in *D. avara* occurred in spring-early summer, broadly coinciding with the highest values of avarol and 5′-monoacetylavarol in the first year, while in the second year embryos were already present while values of metabolites were still low (April and May).

### 2.2. Intra-Individual Variation of Metabolites

**Figure 4:**
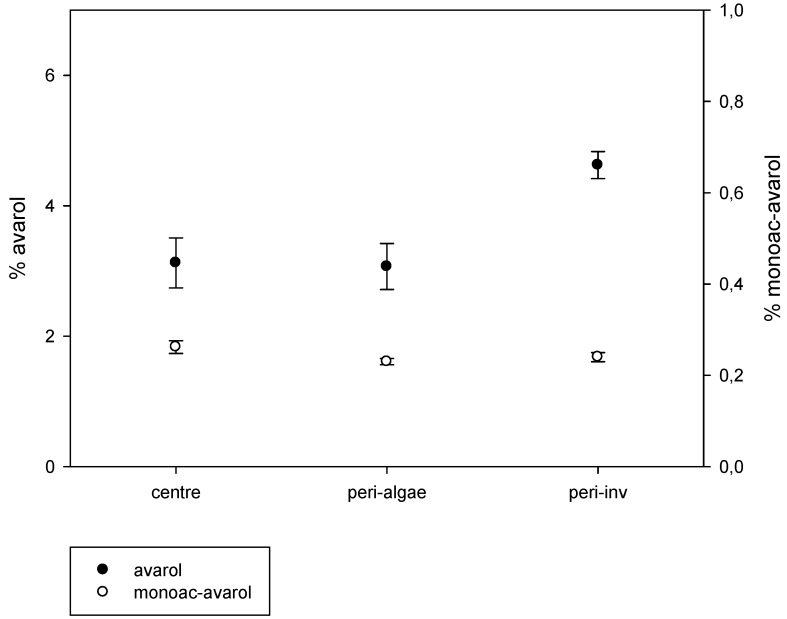
Average percentage of avarol and 5′-monoacetylavarol (mg of the secondary metabolite/mg of sponge dry weight) in the central zone of the sponges, periphery of the sponges in contact with invertebrates, and periphery of the sponges in contact with algae (vertical lines are standard errors).

The results of the intra-sponge variation of metabolites are depicted in Figure 4. We found significant intra-individual variation of avarol production (Kruskal-Wallis test, *p* < 0.001). The peripheral zones in close contact with other invertebrates showed significantly higher avarol concentrations than those in the central part of the sponges and in the periphery in contact with algae (pairwise comparisons, *p* < 0.01). Conversely, there were no significant differences in the amount of avarol in the central zones of the sponges and in the peripheral zones in close contact with algae (pairwise comparisons, *p* > 0.05). The 5′-monoacetylavarol did not present significant differences among the different parts of the sponges (Kruskal-Wallis test, *p* > 0.05) (Figure 4).

**Figure 5:**
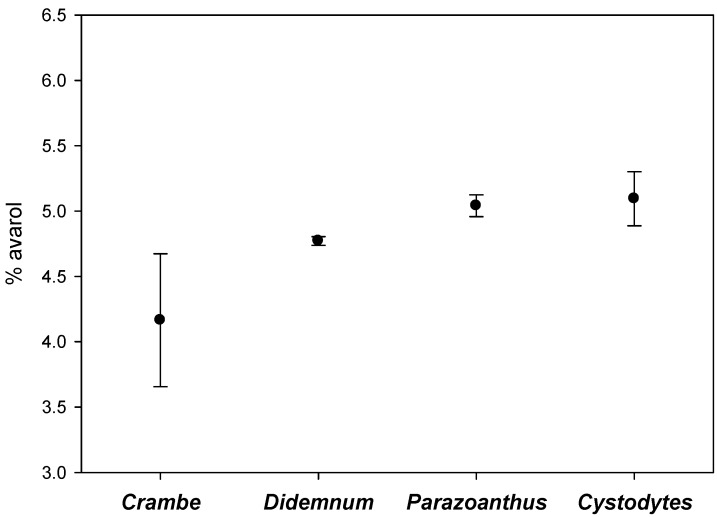
Average percentage of avarol (mg of the secondary metabolite/mg of sponge dry weight) in the periphery of the sponges in contact with *Crambe crambe*, *Didemnum fulgens*, *Cystodytes dellechiajei*, and *Parazoanthus axinellae* (vertical lines are standard errors).

Among the contacts recorded, the commonest were with the sponge *Crambe crambe*, the ascidians *Didemnum fulgens* and *Cystodytes dellechiajei*, and the cnidarian *Parazoanthus axinellae*. The mean avarol concentrations of the sponges’ periphery in close contact with these four different invertebrates are depicted in Figure 5. Although the values for the contacts with *Crambe crambe* were somewhat lower, there were no significant differences among the different invertebrates (Kruskal-Wallis test, *p* = 0.117).

## 3. Discussion

The temporal variation in the production of secondary metabolites in sessile marine invertebrates has often been interpreted (within the Optimal Defence Theory framework [50]) as a trade-off in resource allocation between the organisms’ defence and their primary biological functions such as reproduction and/or growth [17,18,51]. In turn, as temperature often drives investment in reproduction and growth, changes in metabolite production can be correlated with temperature [8,21,52]. In *Dysidea avara*, although both avarol and 5′-monoacetylavarol vary with time, no direct relationship between metabolite production and seawater temperature has been found. Specifically, no relationship existed with current temperature and with temperature in the preceding months. We also found that the highest concentrations of avarol and its monoacetylated derivative roughly coincided with the brooding period (especially during the first study year), so no negative relationship with reproduction could be substantiated, in contrast with findings in other sponges [20]. On the other hand, the minima of metabolite concentration were observed during the autumn of 2009 (October and November) coinciding with the growth reactivation that followed the summer period in the Mediterranean in many invertebrates [53] and in *Dysidea avara* in particular [54]. However, in another sympatric sponge (*Crambe crambe*), the highest values of toxicity were found in autumn [20], and the minimal values of avarol in *D. avara* were not repeated in the second year studied. All this, highlights the need of long-term studies of metabolite variation in a range of species to achieve robust conclusions and also suggests the existence of species-specific factors that could affect the metabolite production in sponges.

The lack of a consistent temporal trend in the concentration of avarol and 5′-monoacetylavarol for the two study years and an inter-individual variation higher than the temporal one, suggested that some external, local factor may determine in some way the avarol production. The induction of secondary metabolite production in sponges has been reported under predation pressure [6], wounding [55], defence against infections [56] and/or overgrowth by other organisms [57]. Moreover, the bioactive compound concentrations can differ both intra- and inter-individually [56,57,58,59]. Particularly, the decoupling between central and peripheral zones of *Crambe crambe* indicated that the sponge defences can be locally induced to some extent [20,60].

For the first time, our results show that sponges are able to produce or accumulate the secondary metabolites in specific parts of their tissue depending on the nature of their neighbors. This finding opens new possibilities to enhance metabolite production in this species for which culture methods have been developed [46,47,48]. 

The higher proportion of avarol in the sponge periphery in contact with other invertebrates than in the sponge central part or in the peripheral zones in contact with algae, points to avarol as a metabolite with an allelopathic role in space competition with long-lived invertebrates. Hard-Bottom communities of the Mediterranean are space-limited systems, where competition for the space with other sessile organisms is one of the main challenges that benthic species must face [59,61,62,63], and allelopathy can give chemically-defended species a competitive edge over other organisms. Our results indicate that space competition can influence the production of secondary metabolites in *Dysidea avara*. The sessile invertebrates found in contact with *D. avara* such as the sponges, ascidians or cnidarians are considered slow growing, long-lived animals mainly competing for the space chemically [52,57,58]. Moreover, the interactions among these sessile organisms are likely to be long-term encounters [64]. Conversely, most algae grow seasonally in the Mediterranean Sea [65,66], thus generating short term interactions with their neighbors that are less prone to be chemically-mediated. There were no significant differences in the effect of the four main invertebrates found in close contact with *D. avara*, although a non-significant trend was observed towards lower production of avarol when in contact with *C. crambe*. Species-specific effects deserve further research given their potential importance in space competition among sessile invertebrates. 

Other functions of avarol, such as predation deterrence, antifouling, or control of infections, *etc.* among others, cannot be excluded, although an anti-predatory function has been signaled as less probable in *D. avara* adults [34]. It may be advantageous to maintain a bioactive metabolite baseline throughout the sponge tissue and to concentrate or produce it in higher amounts on the peripheral borders of the sponge where they can effectively influence the neighbors in close contact. On the other hand, the fact that the 5′-monoacetylavarol presented similar concentrations among the different parts of the sponge tissue indicates that this metabolite probably depends more on intrinsic factors than the avarol does.

## 4. Experimental Section

### 4.1. Sampling

The study was carried out in the locality of l’Escala in the Northwest Mediterranean sea (42°06′52″N, 3°10′07″E). All samples were collected between 10 and 14 m depth from a dense *Dysidea avara* population, sitting on a rocky wall facing NW.

The temporal variability in production of avarol and its monoacetylated derivative was assessed by sampling randomly ten *Dysidea avara* individuals once a month over almost two years (from March 2009 to January 2011). Temperatures were continuously recorded *in situ* by a Stowaway Tidbits^®^, autonomous data logger (0.2 °C precision, 0.15 °C resolution), placed at the study site, at 14 m of depth.

The end of the reproductive period was assessed by verifying the presence of brooded embryos in the sponge tissue through a stereomicroscope. Histological observations by the authors revealed that the reproductive period (*i.e.*, gamete formation) started *ca*. two months before the presence of mature embryos could be detected. 

To study the intra-individual variation in production of avarol and its monoacetylated derivative as a function of the type of organism in contact with the sponge (seaweeds or invertebrates), we sampled 90 *Dysidea avara* individuals in June 2010. As sponges commonly had different contacts along their periphery, we chose a completely random design to ensure independent samples, so only one sample was taken per sponge specimen. We chose 30 individuals featuring contacts with invertebrates, from which we sampled a piece of the contact area, while recording the species in contact with the sponge; we sampled in the same way another 30 sponges in contact with seaweeds and, finally, we took samples from the sponge center of a further 30 individuals. The latter samples were used as a measure of the constitutive (not-induced by the presence of neighbors) amount of avarol.

In all cases, the samples consisted of sponge fragments of *ca*. 8 cm^3^ in volume, comprising the whole sponge thickness. The samples were placed in plastic bags underwater, carried immediately to the laboratory in coolers, cleaned from other materials with forceps, freeze-dried, and kept at −20 °C until subsequent chemical analyses.

### 4.2. Preparation of the Analytical Standards

To obtain pure avarol and its monoacetylated derivative, we collected several specimens of *Dysidea avara* and obtained approximately 6.8 g of freeze-dried material, which was then extracted with a 1:1 (v:v) mixture of dichloromethane and methanol (3 × 50 mL). A total of 1.6 g of crude extract was obtained after filtration and evaporation of the solvent under reduced pressure. The crude extract was subjected to silica gel flash column chromatography using stepwise elutions with heptane and ethyl acetate. The fraction eluted with a mixture of heptane:ethyl acetate 7:3 (v:v) afforded pure avarol (Figure 1) (178 mg) after crystallisation from *n*-hexane/diethyl ether. The spectral data of the isolated compound (UV, MS, and ^1^H and ^13^C NMR) were in full agreement with published values [39].

The 5′-monoacetylavarol (Figure 1) was obtained by partial acetylation of avarol following the protocol described in the literature [40]. The structure of 5′-monoacetylavarol was verified by comparison with the spectral data (UV, MS, and ^1^H and ^13^C NMR) already published for this compound and the position of the acetyl group was confirmed by long-range heteronuclear correlations observable on NMR spectra (HMBC experiments) of 5′-monoacetylavarol. Both 1D and 2D NMR spectra were recorded in DMSO-*d*_6_ on a Jeol EX 400 spectrometer using standard Jeol pulse sequence programs.

### 4.3. Chemical Extraction Procedure

For each sample, we ground 50 mg of freeze-dried sponge and extracted the resulting powder two successive times with 2.5 mL of dichloromethane. At each extraction, the mixture was sonicated for 10 min, and filtered through a 20 μm polytetrafluoroethylene filter (PTFE). The two extracts were pooled, the solvent was evaporated by bubbling through nitrogen and the crude extracts were then re-dissolved in 5 mL of methanol. An aliquot of 1.5 mL was passed through a 0.2 μm PTFE syringe filter before high-performance liquid chromatography (HPLC) injection (10 μL of each sample, two injections per sample).

### 4.4. HPLC Analysis and Quantification

We verified the compounds extracted using a qualitative analysis carried out on a LC/DAD/ESIMS system equipped with an Accela-Thermo Fisher Scientific photodiode array detector and a LCQFleet-Thermo Fisher Scientific mass spectrometer (ion trap) using a Phenomenex Gemini C_6_-phenyl (3 × 150 mm, 5 μm) analytical column with a gradient of water/acetonitrile/formic acid 0.1%. We then performed quantitative analyses of the compounds of interest with a Waters Alliance 2695 Separations Module and Waters 996 photodiode array detector, using a Phenomenex Gemini C_6_-phenyl (3 × 150 mm, 5 μm) analytical column. A linear gradient solvent system consisted of 0.1% trifluoroacetic acid in water (solvent A) and 0.1% trifluoroacetic acid in acetonitrile (solvent B) and graded from 70% (solvent A) and 30% (solvent B) to 0% (solvent A) and 100% (solvent B) in 10 min at the flow rate of 0.5 mL·min^−1^. The retention times of avarol and 5′-monoacetylavarol were approximately 3.6 min and 4.5 min, respectively.

The calibration curves of avarol and 5′-monoacetylavarol were prepared with the isolated sesquiterpenoids used as external standards at 15 concentrations at 0.2 mg·mL^−1^ steps ranging from 0.2 to 3 mg·mL^−1^. The regression equations were calculated as *y* = a*x* + b, where *y* and *x* corresponded to the peak area and compound concentration, respectively. Integration of the peaks corresponding to avarol and 5′-monoacetylavarol was registered at 298 and 282 nm, respectively. The correlation coefficients obtained (*r*) were 0.996 for avarol and 0.998 for 5′-monoacetylavarol. The amount of avarol and 5′-monoacetylavarol in the sample extracts (mg·mL^−1^) was calculated from peak area readings using the corresponding equation. The percentage of avarol and 5′-monoacetylavarol relative to dry weight of the sponge was then calculated for each sample.

### 4.5. Data Analysis

Mass spectrometric data acquisition and processing were performed using Xcalibur-Thermo Fisher Scientific software. All the chemical quantitative analyses were performed using Empower software.

Differences in avarol and 5′-monoacetylavarol concentrations with time (fixed factor) were analyzed by means of a permutational analysis of variance (PERMANOVA) using the Euclidean distance, as implemented in the (Primer v.6 package, Primer E Ltd., Plymouth, UK). The null distribution of the test statistic in PERMANOVA is produced by permutation, thus relaxing the usual normality assumptions of analysis of variance [67]. Pair-wise *a posteriori* comparisons were performed by the program, and the corresponding *p*-values were adjusted for multiple comparisons following the B-Y (Benjamini and Yekutieli) false discovery rate method [68].

Cross-Correlation analyses were also performed to check for relationships between monthly concentrations of avarol and 5′-monoacetylavarol with temperature (Systat v.12 package, Systat Software Inc., Washington, WA, USA). In these analyses, the correlation was calculated by lagging one series with respect to the other. Positive time-lags indicate correlation of the first series of values with the values obtained in subsequent months of the second series, and the reverse is true for correlations at negative time-lags. The correlation at time-lag = 0 is the usual Pearson correlation.

For the analysis of inter-individual variability of avarol and 5′-monoacetylavarol, the non-parametric Kruskal-Wallis test was applied since data did not comply with the assumptions of normality and homogeneity of variances. *Post-hoc* pairwise comparisons were done with Dunn’s test (Statistica v.7 package, StatSoft Inc., Tulsa, OK, USA).

## 5. Conclusions

A natural variation in the amount of avarol appears to be intrinsic to the species but modulated by the nature of the neighbors in close contact, which makes it difficult to outline a consistent temporal pattern. Although more observational and experimental studies have to be addressed to understand the mechanisms involved and to determine the possible contribution of other ecological and physical factors not considered in this study, the influence of the neighbors would explain part of the high variability commonly reported in previous literature. If the neighbors influence metabolite yields, especially at the periphery of the sponge, the type of organisms in close contact with the target species should be considered in temporal surveys. Furthermore, this finding opens new biotechnological approaches to enhance the metabolite supply in sponge cultures by developing experimental settings that incorporate interactions with competing species.
